# PLASMA BIOMARKERS FOR ALZHEIMER’S DISEASE AND RELATED NEUROPATHOLOGY IN RELATION TO MCI AND DEMENTIA: THE ARIC STUDY

**DOI:** 10.1093/geroni/igad104.3716

**Published:** 2023-12-21

**Authors:** Kevin Sullivan, Chad Blackshear, Keenan Walker, Michelle Mielke, B Gwen Windham, Michael Griswold, Thomas Mosley, Priya Palta

**Affiliations:** University of Mississippi Medical Center, Jackson, Mississippi, United States; University of Mississippi Medical Center, Jackson, Mississippi, United States; National Institute on Aging, NIH, Baltimore, Maryland, United States; Wake Forest University School of Medicine, Winston-Salem, North Carolina, United States; UMMC-The MIND Center, Jackson, Mississippi, United States; UMMC-The MIND Center, Jackson, Mississippi, United States; University of Mississippi Medical Center, Jackson, Mississippi, United States; UNC Gillings School of Global Public Health, Chapel Hill, North Carolina, United States

## Abstract

Blood-biomarkers for Alzheimer’s Disease and related neuropathological processes, including amyloidosis, tau deposition, and neurodegeneration, may revolutionize the monitoring of risk and progression of neurocognitive outcomes in older adults. At visit 5 (2011-13) of the ARIC study, a multi-center longitudinal cohort study, amyloid-beta (Aβ42:Aβ40), ptau-181, glial fibrillary acidic protein (GFAP), and neurofilament light (NFL) were measured in plasma using an ultrasensitive single-molecule array (Simoa; Quanterix N4PE + p-tau-181 single-plex) in a sample of 1,711 participants (Age=76±5 years, 60% Female, 28% Black). Neurocognitive outcomes included adjudicated dementia and mild cognitive impairment (MCI) through 2019 for a median follow-up of 5.6 years. Multinomial logistic regressions estimated Relative Risk Ratios (RRR) of the four-category outcome of MCI (n=543), dementia (n=371), and death (n=59) vs. cognitively unimpaired (n=736) for each base(2) log-transformed biomarker, adjusted for age, sex, education, site-race, hypertension, diabetes, BMI, APOE, and eGFR. When estimates appeared similar for MCI and dementia, an additional analysis modeled RRR of a three-category outcome of combined MCI/dementia, death, and cognitively unimpaired. Higher plasma Aβ42:Aβ40 ratio was associated with lower MCI/dementia risk (RRR=0.48, 95%CI: 0.32,0.73). Higher levels of all other biomarkers were associated with higher risk of MCI [p-tau181 (RRR=1.57, 95%CI: 1.23,2.00); GFAP (RRR=1.31, 95%CI: 1.03,1.67); NFL (RRR=1.64, 95%CI: 1.26,2.13)] and dementia [p-tau181 (RRR=3.16, 95%CI: 2.43,4.11); GFAP (RRR=2.40, 95%CI: 1.80,3.20); NFL (RRR=3.62, 95%CI: 2.58,5.09)]. These results indicate blood-biomarkers representative of multiple neuropathological processes were strongly associated with both MCI and all-cause dementia outcomes, emphasizing their potential role as tools in risk identification, early diagnosis, and possibly guiding interventions.

